# Inferior vena cava ultrasound to guide fluid management for prevention of hypotension after spinal anesthesia in a tertiary level teaching hospital

**DOI:** 10.1097/MS9.0000000000004449

**Published:** 2025-11-25

**Authors:** Semanta Dahal, Bishwas Pradhan, Amit S. Bhattarai, Shiva P. Paudyal, Anil Shrestha, Gentle S. Shrestha

**Affiliations:** a Department of Critical Care Medicine, Om Hospital and Research Centre, Chabahil, Kathmandu, Nepal; b Department of Anesthesiology, Manmohan Cardiothoracic Vascular and Transplant Center, Maharajgunj, Kathmandu, Nepal; c Department of Anesthesiology, Tribhuvan University Teaching Hospital, Maharajgunj, Kathmandu, Nepal; d Department of Critical Care Medicine, Tribhuvan University Teaching Hospital, Maharajgunj, Kathmandu, Nepal

**Keywords:** collapsibility index, hypotension, inferior vena cava, spinal anesthesia, transthoracic echocardiography, ultrasound

## Abstract

**Background:**

Hypotension is common during spinal anesthesia and contributes to organ hypoperfusion. Inferior vena cava ultrasonography (IVC USG) has been used in critically ill patients for predicting volume responsiveness but data regarding its use in perioperative setting is limited. This study aims to evaluate the use of IVC USG to guide fluid management for prevention of hypotension after spinal anesthesia.

**Materials and methods:**

In this prospective, randomized, interventional study, 92 patients undergoing surgery under spinal anesthesia were randomized into USG group or Control group. In the USG group, IVC USG assessment and fluid management was performed before spinal anesthesia. In the control group, spinal anesthesia was performed without IVC USG assessment. In both the groups, incidence of hypotension and amount of fluid and vasopressors administered were recorded.

**Results:**

The incidence of hypotension was significantly lower in the USG group 14 patients (30%) when compared to the control group 24 patients (52%; *P* = 0.034). In both the groups, the median vasopressors requirements were not statistically significant (*P* = 0.116). The median amount of fluid administered in USG group was 460 ml while it was 300 ml in control group which was not statistically significant (*P* = 0.185). However, the median of amount of fluid administered after spinal anesthesia was 150 ml in the USG group as compared to 300 ml in the control group, which was statistically significant (*P* < 0.001).

**Conclusion:**

IVC USG is an effective modality for optimization of volume status for prevention of hypotension after spinal anesthesia.

## Introduction

Hypotension is a frequently observed side effect of spinal anesthesia^[1,2]^. It is attributed to reduction in both cardiac output and systemic vascular resistance due to local anesthetic induced sympathetic blockade^[3,4]^. Even short duration of intraoperative mean arterial pressure (MAP) less than 55 mmHg has been found to be associated with acute kidney injury (AKI) and myocardial injury^[5–7]^. Severe episodes of intraoperative hypotension is an independent risk factor for myocardial infarction, stroke, heart failure, AKI, prolonged hospital stay, and increased 1-year mortality rates^[8,9]^.

Preoperative volume status is an important factor determining patient’s hemodynamic status^[10]^. The incidence of hypotension during spinal anesthesia ranges from 25 to 75%. Commonly used methods for prevention of hypotension in spinal anesthesia include fluid preloading and/or co-loading with or without vasopressors^[1,2]^. However, empiric preloading carries risk of fluid overload especially in elderly and cardiac patients^[11]^. Furthermore, prophylactic empiric volume loading to prevent spinal anesthesia induced hypotension has provided inconsistent results in various studies^[12,13]^. Though commonly practiced in obstetrics population, empiric prophylactic volume loading is not routinely performed in other patients undergoing spinal anesthesia^[14]^.

Accurate assessment of intravascular volume is challenging. Central venous pressure guided fluid management is invasive and has been demonstrated to be less reliable^[15,16]^. Similarly, use of pulmonary capillary wedge pressure (PCWP) is invasive, exposes patient to potential harm and has not shown to be reliable either for determining volume status or responsiveness for resuscitation. Dynamic parameters like inferior vena cava diameter and collapsibility index (CI), echocardiography, stroke volume variation and pulse pressure variation are being used widely for assessing volume status. Stroke volume variation and pulse pressure variation are invasive and require sedated and mechanically ventilated patients^[16,17]^.

Inferior vena cava ultrasonography (IVC USG) is noninvasive, quickly learned and performed and can be used in perioperative setting^[16,18]^. It has been used for prediction of hypotension after induction of general anesthesia^[8,19]^. It has been recently used for prevention of hypotension in spinal anesthesia by guiding fluid management^[20]^ and has also been used to predict hypotension after induction of spinal anesthesia^[21]^.

This study aims to evaluate the use of IVC USG guided volume optimization for prevention of hypotension after spinal anesthesia.

**HIGHLIGHTS**

- Inferior vena cava ultrasound (IVC USG) can be used to optimize volume status.
- IVC USG guided fluid therapy is associated with lower incidence of hypotension.
- Such strategy can also be associated with reduced used of vasopressors.

## Methods

### Study design and settings

**Figure 1.**
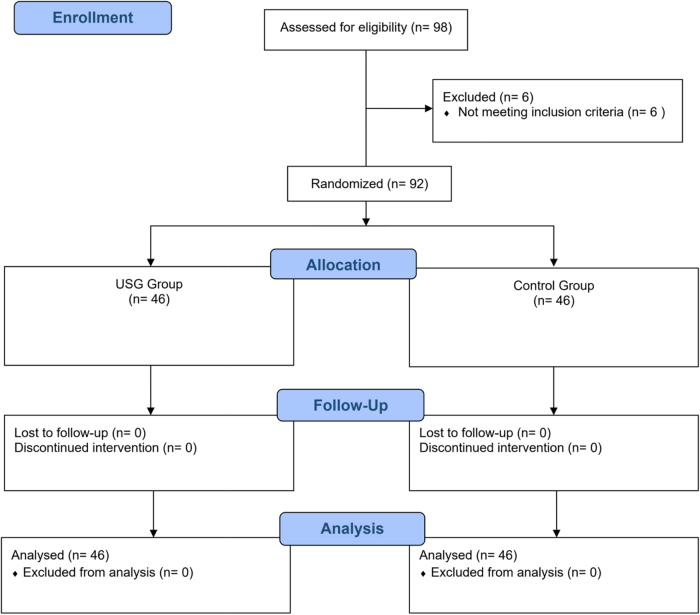
CONSORT flowchart.

This was a randomized controlled study conducted in accordance with the Declaration of Helsinki, from December 2018 to September 2019. The study was conducted in accordance with the CONSORT guidelines. This study was approved by the institutional review committee of Institute of Medicine (IOM; No. 308/075/076) on 10 December 2018 and was registered in clinicalTrials.gov (NCT04736498). We enrolled 92 American Society of Anesthesiology physical status (ASA PS) I and II patients aged 16–65 years requiring elective spinal anesthesia for lower limb orthopedic surgery (Fig. 1). Exclusion criteria were patients with pre-procedural hypotension, defined as two consecutive measurements of systolic blood pressure (SBP) less than 90 mmHg or MAP less of 60 mmHg or with contraindication for spinal anesthesia. Written informed consent was obtained from the patient a day prior to surgery during pre-anesthetic assessment. Premedication was done with diazepam 10 milligram (mg) PO before bedtime if patient’s weight was ˃50 kilogram (kg) and 5 mg if weight was ≤50 kg. Randomization was done using computer generated sequence into two groups: USG group (Group U) and Control group (Group C). We enrolled 46 cases in each group. None of the patients were lost to follow-up and analysis (Fig. 1). At pre-anesthetic preparation room baseline noninvasive blood pressure measurements [SBP, diastolic blood pressure (DBP), MAP], heart rate (HR) and peripheral oxygen saturation (SpO_2_) were recorded. An 18Gauge intravenous access was secured and a Ringer’s lactate drip was attached. No pre-loading was done to any patient. In the IVC USG group, all patients were kept supine for at least 5 minutes prior to IVC examination. Ultrasound measurements was performed using a Sonosite M-Turbo (Sonosite Inc., USA) machine and phased array 5–1 MHZ transducer (Sonosite Inc.). Assessment of IVC was performed as described in the study by Ceruti *et al*^[20]^. Collapsibility index (CI) was calculated from the values of maximum and minimum IVD diameter. All IVC measurements were performed by principal investigator before spinal anesthesia. Principal investigator had performed more than 25 supervised scans before the commencement of the study.

A CI of ˃36% was accepted as predicted fluid responder and ≤36% was regarded as predicted fluid non-responders. Predicted fluid responders were given a bolus of 500 ml of Ringer’s lactate over 15 minutes, after which the CI was reassessed. Additional 250 ml of Ringer’s lactate bolus was given until a non-fluid responder pattern was observed. Then, patient was shifted to operation theatre and spinal anesthesia was performed. Control group followed the standard procedure in our center and did not undergo USG assessment and did not receive fluid before spinal anesthesia. Spinal anesthesia was performed using 3 ml of hyperbaric bupivacaine 0.5% in sitting position. Meanwhile, the noninvasive blood pressure and heart rate was measured and recorded at 0 minutes, then every 3 minutes for 30 minutes, which was the study period and then every 5 minutes throughout surgery and anesthesia. An infusion of Ringer’s lactate at 4 ml/kg/hr was administered during the procedure. Total amount of fluid administered was recorded before and after spinal anesthesia. Degree of sensory block was assessed for loss of cold sensation by cold cotton swab and motor block was assessed using modified Bromage scale^[22]^. Hypotension was defined as a drop in SBP of either an absolute value of systolic pressure <90 mmHg or an absolute value of MAP < 60 mmHg or a reduction in MAP >30% of the baseline value (baseline values assessed in the pre-anesthetic preparation room). In case of hypotension, patient was managed with 4 ml/kg of Ringer’s lactate, infused over 5 minutes. If hypotension persisted after initial fluid bolus, intravenous mephentermine 6 mg was given. If hypotension was not corrected, a second dose of intravenous 6 mg mephentermine was repeated after 3 minutes. Intravenous phenylephrine 100 µg was given as rescue drug if the patient did not respond to initial fluid bolus and 12 mg of mephentermine. Bradycardia was defined as HR less than 45 bpm was treated with intravenous atropine 0.6 mg. The primary outcome was comparison of incidence of hypotension and secondary outcomes were use of fluids and vasopressors in the two groups.

### Sample size

We assumed the event rate of 57.9% (hypotension in the control group) as reported by Chinachoti *et al*^[2]^ and a 30.4% absolute reduction of the hypotension with intervention with the resultant incidence of 27.5% as reported by Ceruti *et al*^[20]^.

We considered 95% confidence interval with the power of 80%, dropout rate of 10% and significance level of 5% for our study, with the resultant sample size of 46 in each study group.

### Data collection and statistical analysis

Data collection was done in a preformed proforma. Statistical analysis was done by using statistical package for the social sciences (SPSS) software version 29 (SPSS Ltd, Chicago, IL, USA). Continuous variables were as mean (±standard deviation) and categorical variables were reported as numbers (with percentage). Hemodynamic parameters were analyzed using independent sample *t*-test for intergroup comparison. Categorical data were analyzed with chi square test. Paired *t*-test was used for normally distributed dependent variable and Mann–Whitney *U* test was used for non-normally distributed parameters. For all determinations, *P*-value <0.05 was considered statistically significant. A multivariable logistic regression analysis was conducted to assess relationship of variables with occurrence of hypotension. Covariates were adjusted.

## Results

The mean age of our study population was 38.67 ± 15.20 years. There was no significant difference between the groups regarding age, weight, sex, and ASA PS (Table 1). The baseline hemodynamic parameters (HR, SBP, DBP, and MAP) were also similar between the groups (Table 2).

**Table 1:** Demographic profile

Parameter	USG group (*n* = 46)	Control group (*n* = 46)	*P*-value	95% Confidence interval for difference of means
	Mean ± SD	Mean ± SD		
Age in years	37.58 ± 15.25	39.76 ± 15.48	0.499	4.19–8.54
Weight in kg	61.76 ± 9.00	62.48 ± 9.01	0.711	3.04–4.43
	**Frequency (%)**	**Frequency (%)**	***P*-value**	
Sex				
Male	30 (65)	33 (72)	0.501	
Female	16 (35)	13 (28)		
ASA PS				
ASA I	30 (65)	28 (61)	0.666	
ASA II	16 (35)	18 (39)

**Table 2:** Comparison of baseline hemodynamic parameters

Baseline	USG group (*n* = 46)	Control group (*n* = 46)	*P*-value
	Mean ± SD	Mean ± SD	
HR in bpm	79.19 ± 15.60	74.48 ± 24.12	0.268
SBP in mmHg	128.47 + 14.33	131.52 + 15.65	0.333
DBP in mmHg	80.5 ± 8.38	81.69 ± 8.42	0.497
MAP in mmHg	95.69 ± 9.68	99 ± 11.16	0.130

**Figure 2.**
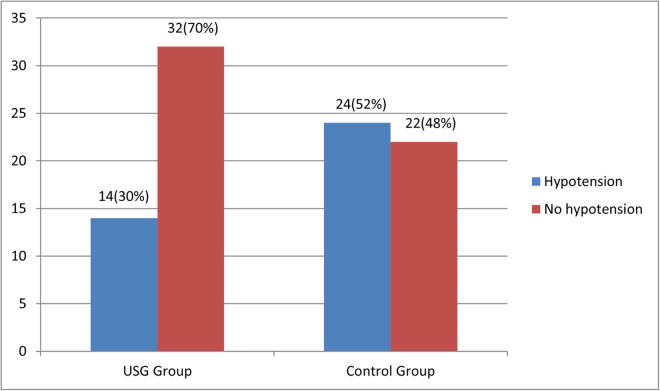
Incidence of hypotension.

The overall incidence of hypotension in our study was 41.3% (*n* = 38). The incidence of hypotension in the control group was found to be 52% (*n* = 24) compared to 30% (*n* = 14) in USG group which was found to be statistically significant (*P* = 0.034; Fig. 2).

Comparison of HR, SBP, DBP, and MAP were done at different time points. There was statistically significant lower MAP in USG group at 3 minutes as compared to control group and statistically significant lower heart rate at 24 minutes in USG group as compared to control group. At all the other times, there was no significant difference in hemodynamic parameters between the two groups (Supplemental Digital Content Table 1, available at: http://links.lww.com/MS9/B36). One patient in control group had an episode of bradycardia managed with intravenous Atropine 0.6 mg.

The median total volume of fluid administered in control group was 300 ml (200–450 ml) and it was 460 ml (150–650 ml). The difference was not statistically significant (*P* = 0.185). However, volume of fluid administered after spinal anesthesia was 200 ml (300–450 ml) in the control group and 150 ml (150–300 ml) in the USG group. The difference was statistically significant (*P* < 0.001). Regarding the dose of vasopressor administered, there was no difference in the median dose of mephentermine administered (*P* = 0.116; Table 3).

**Table 3:** Comparison of fluid and vasopressors used between USG group and control group

Total fluid	Median (Q1, Q3), in ml	*P*-value
Control group	300 (200, 450)	0.185
USG group	460 (150, 650)	
**Post spinal fluid**	**Median (Q1, Q3), in ml**	***P*-value**
Control group	300 (200, 450)	< 0.001^a^
USG group	150 (150, 300)	
**Vasopressor use**	**Median (Q1, Q3), in mg**	***P*-value**
Control group	0 (0, 6)	0.116
USG group	0 (0, 0)	

^a^ Statistically significant (*P* < 0.05)

Q1, 25th percentile

Q3, 75th percentile

Mean difference between CI 1 and CI 2 was 16.44 ± 7.61 which was statistically significant (*P* < 0.001) with 95% CI of 12.88–20.01. There was strong and significant correlation between CI and total fluid administered (Pearson correlation coefficient = 0.829; *P* < 0.001) with 95% CI of 0.71–0.90 (Supplemental Digital Content Figure 1, available at: http://links.lww.com/MS9/B34). Similarly, there was a significant correlation between CI and delta MAP (difference between baseline MAP and MAP at 3 minutes expressed as percentage: baseline – 3 minutes /baseline * 100%) with Pearson correlation coefficient of 0.318; *P* = 0.031; and 95% confidence interval of 0.30–0.557 (Supplemental Digital Content Figure 2, available at: http://links.lww.com/MS9/B35).

However, in multivariable binary logistic regression analysis, considering occurrence of hypotension as the outcome measure, none of the tested variables (age, sex, ASA PS, fluid bolus, baseline SBP, DBP, MAP, and HR) were significant. All the covariates were adjusted.

## Discussion

In this study, IVCCI guided fluid therapy led to 22% reduction in the incidence of hypotension after spinal anesthesia. In a study by Zhang *et al*^[8]^, CI was an independent predictor of hypotension with the odds ratio of 1.17 (1.09–1.26). They concluded that preoperative ultrasound IVCCI measurement was a reliable predictor of hypotension after induction of general anesthesia, wherein CI greater than 43% was the threshold. In a similar prospective observational study by Szabo *et al*^[19]^, 102 patients were recruited to understand the role of inferior vena cava collapsibility index in the prediction of hypotension associated with general anesthesia. The Receiver-operating characteristic curve (ROC) curve analysis for IVCCI showed an area under the curve (AUC) of 64.8% (95% confidence interval: 52.1–77.5%). The positive predictive value was 75.0% (95% confidence interval: 50.9–91.3%), and the negative predictive value was 71.4% (95% confidence interval: 58.7–82.1%). They concluded that in spontaneously breathing preoperative noncardiac surgical patients, preoperatively detected IVCCI ≥50% can predict post induction hypotension with high specificity but low sensitivity. Despite moderate performance, IVCCI is an easy, noninvasive and attractive option to identify patients at risk and should be explored further.

Fluid loading based on preoperative assessment of volume status is one of the common methods used to prevent hypotension. Preoperative volume status is an important factor determining patient’s hemodynamic status and is predictive of hypotension during spinal anesthesia^[10]^. Various invasive and noninvasive techniques has been described for assessment of volume status^[15,16]^.

IVC USG is noninvasive, easy to learn and perform and a validated tool currently being extensively used in various emergency and critical care settings^[16,18,23–27]^. The availability of portable USG devices has made point of care ultrasound (POCUS) like IVC USG very much feasible even in resource limited settings^[28]^. In a systematic review and meta-analysis by Zhang *et al*^[26]^, a total of 8 studies involving 235 patients were analyzed. The cut-off values of ΔIVC varied across studies, ranging from 12% to 40%. The pooled area under the receiver operating characteristic curve was 0.84 (95% confidence interval: 0.79–0.89). They concluded that ΔIVC measured with point-of-care ultrasonography is of great value in predicting fluid responsiveness.

In a prospective observational study by Salama *et al*^[21]^, 100 patients undergoing surgery under spinal anesthesia were included to evaluate the efficacy of both pre-operative inferior vena cava collapsibility index (IVCCI) and inferior vena cava to aorta diameter (IVC: Ao) index for predicting post spinal anesthesia hypotension. 45% patients developed post spinal anesthesia hypotension. IVCCI was significantly higher in patients who developed post spinal anesthesia hypotension than in patients who did not, while IVC: Ao index was significantly lower in patients who developed post spinal anesthesia hypotension than in patients who did not. The ROC curve analysis showed that IVC: Ao index had a sensitivity of 96%, a specificity of 88%, and an accuracy of 95% to predict post spinal anesthesia hypotension at a cut-off point less than 1.2. IVCCI had a sensitivity of 84%, a specificity of 77%, and an accuracy of 84% to predict post spinal anesthesia hypotension at a cut-off point more than 44.7%. Logistic regression analysis revealed that IVCCI and IVC: Ao index were good predictors of the occurrence of post spinal anesthesia hypotension.

In our study we found absolute risk reduction in hypotension of 22% between the groups (USG group 30% vs Control 52%; *P* = 0.034) number needed to treat (NNT) from our study was 4.6. This observation is in line with the findings of Ceruti et al^[20]^. They conducted a prospective, randomized, cohort study in which 160 patients, scheduled for surgery under spinal anesthesia were included. They were randomized in to a study group (IVC USG-group), consisting of an IVC USG analysis before spinal anesthesia with IVC USG-guided volume management and a control group (group C) with no IVC USG assessment. They observed 15% reduction in hypotension in the USG group with NNT of 6.7 (IVC USG-group 27.5%, Group C 42.5%; *P* = 0.044).

We found that the median of dosage of vasoactive drugs in control group when compared to USG group was (0 mg vs 0 mg, *P* = 0.116) which was not statistically significant in our study. However, in a study by Ceruti *et al*^[20]^, it was found that the need for vasoactive drugs in the C-group was significantly higher than in the IVC USG-group (25% vs 12%, *P* = 0.015). This finding was not found to be statistically significant in our study which may be because our study was not powered to assess vasopressor use.

In our study, the median volume of total fluids required during the first 30 minutes of anesthesia was not significantly different between USG and control group (460, 150–650 ml vs 300, 200–450 ml; *P* = 0.185). Ceruti *et al*^[20]^ found the total fluid to be higher in the USG group (665 ml vs 350 ml, *P* = 0.0001). However, the median amount of fluid required after spinal anesthesia was lower in the USG group when compared to the control group (150, 150–300 ml vs 300, 200–450 ml; *P* < 0.001), which was similar to that found by Ceruti *et al*^[20]^. Thus, our study found that requirement of fluid in post spinal anesthesia state was lower in USG group compared to control group, this finding indicated adequate fluid optimization prior to induction of spinal anesthesia.

Our study also showed a significant correlation between CI and total fluid needed that may be partially due to our study’s protocol design. Ceruti *et al*^[20]^ demonstrated similar findings in their study. Similarly, a weak but significant correlation was found between CI and delta MAP in our study which was similar to study by Zhang *et al*^[8]^ where CI was also positively associated with a percentage decrease in MAP (*r* = 0.27).

However, in multivariable logistic regression analysis, we did not find any of the tested covariates to be independently associated with occurrence of hypotension. Though IVC USG is a feasible bedside tool, it has some inherent limitations for prediction of fluid responsiveness in spontaneously breathing patients. Also, hypotension after spinal anesthesia is multifactorial. Future studies comparing IVC USG with other modalities for predicting fluid responsiveness during spinal anesthesia and testing myriad of factors that can be associated with occurrence of hypotension can be beneficial^[29,30]^.

## Limitations

Our study has few limitations. Ultrasonography is operator dependent technique and may have some inter-user variability in measurement. Similarly, the breathing pattern of the patient may affect the measurement of IVC and might alter the findings. Likewise, due to the inherent nature of the study patient blinding was not feasible. USG findings between two groups were not compared as we did not assess IVC in the control group. Further studies with assessment of IVC in control group might provide more useful findings. Our study was done on ASA 1 and 2 patients only, so further studies are needed for generalization of findings to other settings like emergency surgery, elderly, high-risk patients, etc. Similarly, we did not record any adverse effects in perioperative period.

## Conclusion

IVC USG is a noninvasive and an effective modality that can be used for optimization of volume status and for prevention of hypotension after spinal anesthesia. Further studies will be needed for assessing IVC USG usefulness in elderly and high-risk hemodynamically unstable patients.

## Data Availability

The data that support the findings of this study are available from the corresponding author, GSS, upon reasonable request.

## Research registration unique identifying number (UIN)

The study was registered in ClinicalTrials.gov (NCT04736498).
